# Navigating the brain: the role of exosomal shuttles in precision therapeutics

**DOI:** 10.3389/fneur.2023.1324216

**Published:** 2024-01-18

**Authors:** Shaheera Fatima, Ariba Qaiser, Saadia Andleeb, Asraf Hussain Hashmi, Sobia Manzoor

**Affiliations:** ^1^Atta-ur-Rehman School of Applied Biosciences, Healthcare Biotechnology, National University of Science and Technology, Islamabad, Pakistan; ^2^Atta-ur-Rehman School of Applied Biosciences, Industrial Biotechnology, National University of Science and Technology, Islamabad, Pakistan; ^3^Institute of Biomedical and Genetic Engineering (IBGE), Islamabad, Pakistan

**Keywords:** BBB, central nervous system (CNS), therapeutic targets, exosomes, drug delivery

## Abstract

Brain diseases have become one of the leading roots of mortality and disability worldwide, contributing a significant part of the disease burden on healthcare systems. The blood-brain barrier (BBB) is a primary physical and biological obstacle that allows only small molecules to pass through it. Its selective permeability is a significant challenge in delivering therapeutics into the brain for treating brain dysfunction. It is estimated that only 2% of the new central nervous system (CNS) therapeutic compounds can cross the BBB and achieve their therapeutic targets. Scientists are exploring various approaches to develop effective cargo delivery vehicles to promote better therapeutics targeting the brain with minimal off-target side effects. Despite different synthetic carriers, one of the natural brain cargo delivery systems, “exosomes,” are now employed to transport drugs through the BBB. Exosomes are naturally occurring small extracellular vesicles (EVs) with unique advantages as a therapeutic delivery system for treating brain disorders. They have beneficial innate aspects of biocompatibility, higher stability, ability to cross BBB, low cytotoxicity, low immunogenicity, homing potential, targeted delivery, and reducing off-site target effects. In this review, we will discuss the limitations of synthetic carriers and the utilization of naturally occurring exosomes as brain-targeted cargo delivery vehicles and highlight the methods for modifying exosome surfaces and drug loading into exosomes. We will also enlist neurodegenerative disorders targeted with genetically modified exosomes for their treatment.

## Graphical Abstract

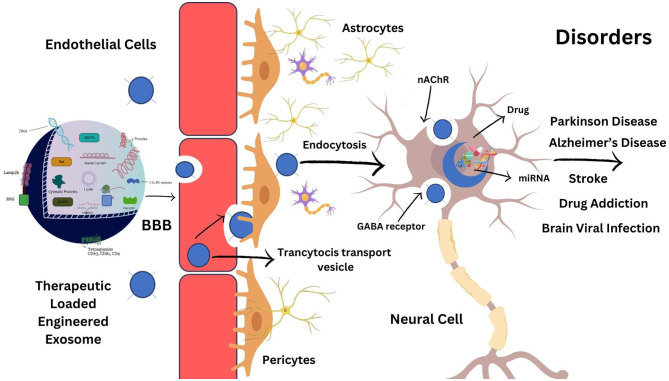

## Introduction

Efficient therapeutics delivery to the brain represents a formidable challenge in treating brain diseases and is one of the most complex areas of pharmaceutical research (1). The brain is protected by a thick barrier separating it from the blood supply and other internal organs (2). The central nervous system (CNS) is guarded by several obstacles, such as the blood-brain barrier (BBB), the blood-CSF (cerebrospinal fluid) barrier, the spinal cord blood, and the blood-retina barrier (3). The permeability of these barriers varies, but the BBB is the broadest and most constrained barrier (4). Comprising continuous capillary endothelial cells, a basement membrane, and a discontinuous stratum of pericytes and astrocytes, the BBB serves as a vital physical barrier (5). It safeguards the brain against potentially harmful substances in the bloodstream by inhibiting their accumulation and transportation into the brain (6). Only small lipophilic compounds weighing <400 Da selectively pass across the BBB (7). This BBB hindrance has blocked the entry of almost 100% of all macromolecules, including recombinants peptides, protein, antibodies, gene therapies, plasmids, and RNAs into the brain, and <98% of all small therapeutic molecules are also unable to cross the BBB. Due to this hindrance, the direct delivery of medicines becomes challenging against neurological diseases like Parkinson's disease, Alzheimer's disease, multiple sclerosis, stroke, brain cancer, and microbial infections (8). Therefore, there is a pressing medical requirement for developing a delivery system capable of traversing the BBB to cure various brain disorders (2).

In recent decades, scientists have focused on developing nanotechnology-based methods to bypass the BBB and target the brain (9). Different types of nanoparticles, including lipid nanoparticles, Liposomes, silica nanoparticles, polymeric nanoparticles, gold nanoparticles, and carbon nanotubes, have all been studied for drug delivery over the BBB (10), particularly for the treatment of brain illnesses such as Alzheimer's and Parkinson's disease (11). Despite of being popularity, the clinical applications of these systems also face challenges (12), such as biocompatibility, poor biodistribution, cell targeting, short half-life in body fluid, poor efficacy while crossing the BBB, inherent immunogenicity, cytotoxicity of carriers and their breakdown products, fast renal clearance by the reticuloendothelial system, and the buildup of nanomaterials like polymers in the brain after multiple administration. These challenges can be overcome by shifting to a naturally developed therapeutic delivery system (13).

Compared to synthetic carriers, natural drug delivery vehicles demonstrate excellent compatibility with intricate biological milieu and can reach the desired tissue or cells more efficiently. This compatibility is expected to boost therapeutic impact while decreasing side effects (14). Exosomes, naturally occurring vehicles, have been introduced to research in recent years to develop therapeutic delivery systems with fewer side effects and improved targeting (15). Although plenty of data have supported the biological functionality and therapeutic value of inherent exosomes and their properties, a detailed review focusing on exosome-based vehicles for organ-specific delivery needs to be included in the literature. Specifically, genetic modification of exosomes for precise delivery to the brain remains an understudied area of research that requires further investigation.

To address this gap in the literature, this review will first present a general overview of exosomes' function and composition, followed by a brief discussion of surface modification techniques and methods of cargo loading into the exosomes. Subsequently, this review provides details on the ability of exosomes to deliver therapeutic agents to the brain, aiming to treat various brain disorders. In addition, the study also discusses current challenges and offers future perspectives for better outcomes.

## Exosome

An exosome is a small-sized subtype of extracellular vesicle (10) connected to endosomal biogenesis pathways. The estimated diameter of exosomes is 30–150 nm and is released by exocytosis (16). Additionally, when examined under transmission electron microscopy, it exhibits a distinctive cup-like appearance (17).

Exosomes serve as vital mediators of cell-cell communication vehicles that deliver nucleic acids, proteins, and lipids, to recipient cells and have been related to various healthy and pathological functions (18).

### Exosome composition

Exosome has a bilayer of lipids that form a nano-spherical membrane-type structure. Additionally, it comprises several kinds of proteins and lipids that come from the original cell from which the exosome is generated. ExoCarta, an exosome database, reports that ~8,000 proteins and 194 lipids have been identified and associated with exosomes to date (19). Its surface is composed of various proteins such as fusion proteins, transport proteins (annexins and flotillin), transmembrane proteins such as tetraspanins CDs protein (CD91, CD63, and CD9), Lamp2b, antigen-presenting molecules, glycoproteins, adhesion molecules, phospholipases, and other lipid-related protein (20). In addition to their protein content, exosomes are abundantly enriched with various lipid components, like phosphoglycerides, sphingolipids, cholesterol, ceramides, and short and long saturated fatty acid chains (21). Exosomes are often characterized according to their size or by surface marker proteins. Tetraspanins, specifically CD9, CD63, and CD81, are widely recognized as the predominant marker proteins found on the surface of exosomes. Tetraspanin antigens are candidates for exosome identification and separation (22).

**Figure 1:**
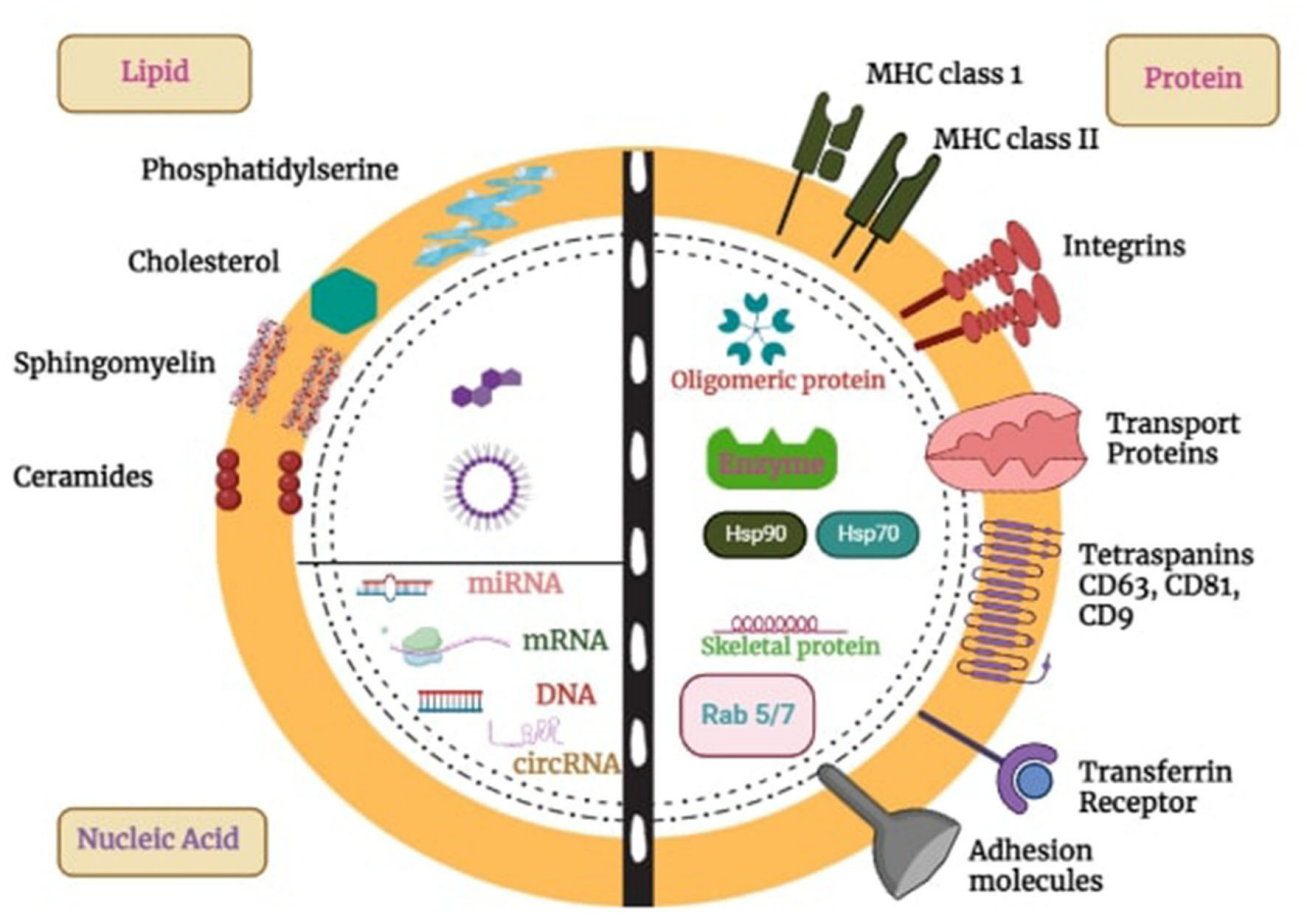
Exosome structure, composition, and cargo. Exosomes, which are produced by all cells, are made up of a lipid bilayer membrane loaded with lipids, transmembrane proteins, and glycoproteins. They have different surface proteins that allow them to be taken up by cells. These vesicles package and transport proteins, DNA, RNA, and other molecules to target cells, influencing a variety of biological functions. This capability makes them promising tools for targeted drug delivery in treating diseases like cancer, brain disorders, etc.

Exosomes encapsulate diverse cargoes, encompassing proteins, nucleic acids, lipids, and other cellular components, as depicted in Figure 1. These cargoes are selectively sorted and packaged into exosomes for targeted delivery to specific cells or tissues (23). Exosomal packaged proteins include enzymes, growth factors, and signaling molecules capable of influencing cellular processes such as differentiation, proliferation, and migration. Exosomes also contain lipids that can influence membrane fluidity and cell signaling pathways. Additionally, they carry different types of RNA molecules, which can affect gene expression and contribute to various physiological and pathological processes (24). The specific cargo composition of exosomes depends on the cell type and conditions.

### Exosome biogenesis and secretion

**Figure 2:**
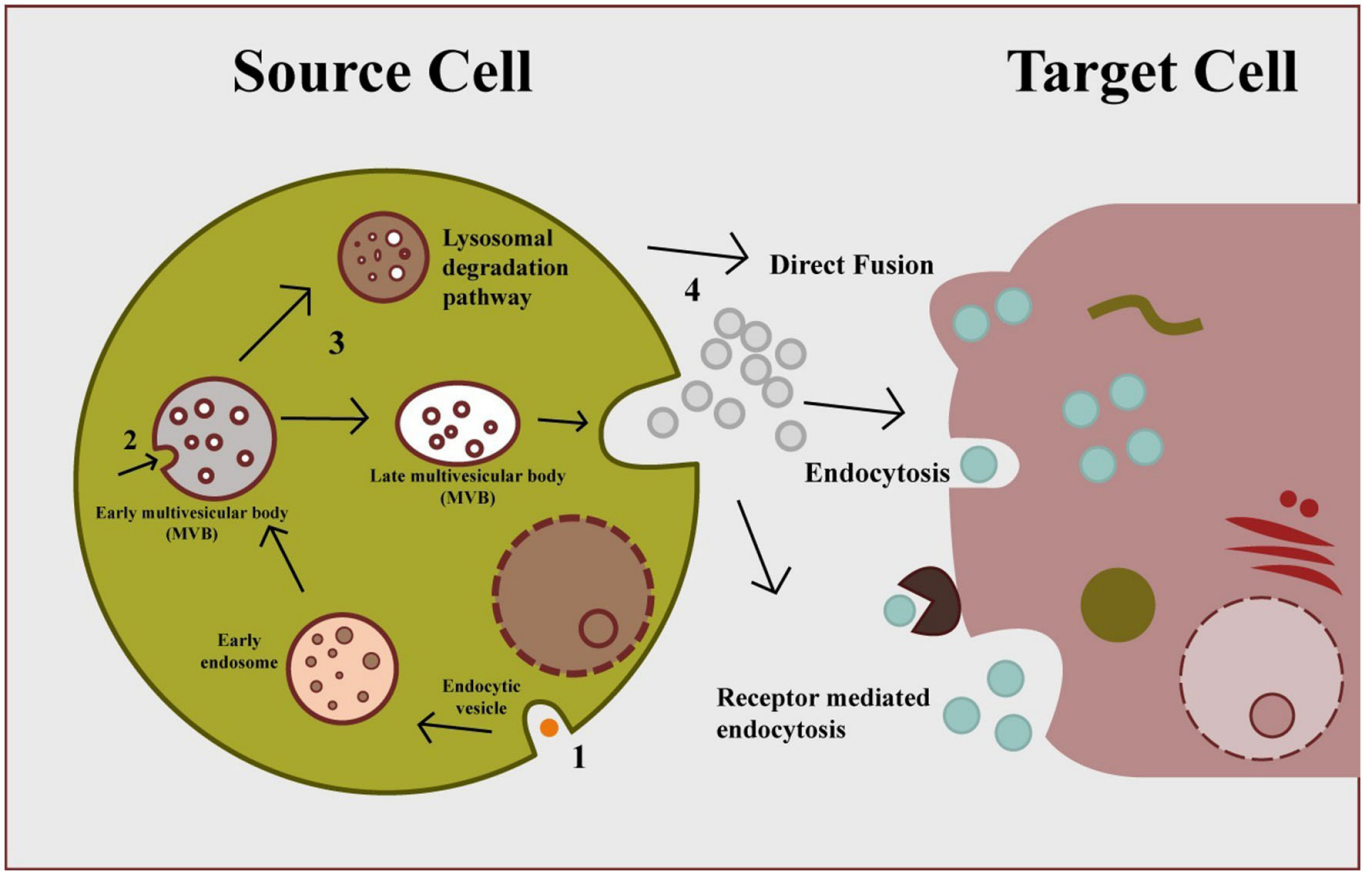
Exosome biogenesis, secretion, and cellular entry. Early endosomes formed by plasma membrane invagination form multivesicular body via inward budding, which secrete exosomes through exocytosis. Secreted exosomes can be uptake by cell via three different mechanisms: direct fusion, endocytosis, and receptor-mediated endocytosis.

Exosomes are produced via the endocytic cellular pathway, which has three stages: early endosomes, late endosomes, and multivesicular bodies (MVBs). Figure 2 depicts the three stages of exosome biogenesis and secretion: plasma membrane invagination produces early endocytic vesicles (early sorting endosomes) with distinct biomarkers on their surfaces, like RabGTPases (25). Along with cell-surface protein, some extracellular contents may also enter the ESEs during this process. After then, the ESEs mature into-late-sorting endosomes. The late limiting membrane can invaginate inward to form Intraluminal vesicles (IVs), which assemble in the late endosome lumen (26). The accumulation of certain cargoes such as protein, lipids, or nucleic acid into ILs via ESCRT-dependent or ESCRT-independent processes takes place during the second step (26). The late endosome is also called a multivesicular body because they accumulate many IVs. The final stage of this process involves the fusion of these multivesicular bodies with either lysosomes/autophagosomes for degradation or may fuse with the plasma membrane to secrete ILs into extracellular space and released ILs are exosome (27). It has been proposed that the exosome biogenesis and secretion have been linked to ESCRT (endosomal sorting complexes) proteins, lipid compounds, the Rab-GTPase family, phospholipids, SNARE (soluble NSF attachment protein receptors), TSG101 (tumor susceptibility gene 101), syndecan-1, and tetraspanins. The entry of secreted exosomes into a recipient call can occur through different channels including direct fusion with the plasma membrane of recipient cells, receptor-mediated endocytosis, and can also take up via phagocytosis, pinocytosis, and endocytosis, which are all mediated by lipid rafts, caveolin or clathrin (28).

### Exosomes surface modification for targeted delivery

Exosome characteristics are inadequate for targeted drug delivery and accumulation in desired tissues. Evidence suggests that exosomes require targeting approaches for therapeutic delivery into recipient cells. However, exosomes' surface can easily be altered. By altering the surface characteristics of exosomes, it is possible to achieve the desired properties that enable engineered exosomes to interact with specific cells (29). For promoting targeting delivery, exosomes can be engineered to express various targeting moieties or ligands, or homing peptides fused with transmembrane protein, acting as Marker protein, on the surface of the exosome (30). This engineered process involves direct modification methods such as physical and chemically changing exosomal surfaces with covalent/non-covalent bonds or indirect methods involving genetic engineering of exosome surface (31). After being engineered, these targeted exosomes are systemically administered, selectively binding to receptors on targeted cells (32). An overview of exosome surface engineering via chemical, physical, and biological is summarized in Table 1.

**Table 1:** Exosome surface engineering techniques for therapeutic delivery.

**Surface engineering categorization**	**Classification**	**Approach**	**Homing molecules**	**Target cell**	**References**
Direct exosome surface engineering	Chemical modification-covalent	PEGylation	(AA-PEG)	Lung carcinoma that overexpresses Sigma receptor	(33)
		Click chemistry	RGE peptide (RGERPPR) for neuropilin-1 targeting	Glioma	(34)
	Chemical modification-non-covalent	Receptor-ligand interaction	The conjugate of superparamagnetic nanoparticles with transferrin	Cancer targeting in the presence of external field	(35)
		Electrostatic interaction	pH-dependent fusogenic peptide/cationic lipids	Increase internalization via endocytosis	(36)
		Hydrophobic insertion	CHP (CSTSMLKACcoupled) with DOPE-NHS linker	Heart	(37)
	Physical modification- exosome-liposome Fusion	Freeze-thawing cycling	PEG-DOPS	HeLa cells	(38)
Indirect surface engineering of exosomes	Genetic modification	The N-terminus of Lamp2b is fused with the target ligand	RVG; muscle-specific peptide	Targeting brain or muscle cells	(39)
		Fusion protein with the PDGFR transmembrane domain	GE11 peptide	Targeting breast cancer cells	(40)
		Fusion protein with the N terminus of Lamp2b	Cardiomyocytes specific peptide	Targeting cardiac tissue	(41)

AA-PEG, Aminoethyl anisamide-PEGlyated co-polymer; CHP peptide, Cardiac homing peptide; DOPE-NHS linker, dioleoylphosphatidylethanolamine N-hydroxysuccinimide; PEG-DOPS, Polyethylene glycol-dopamine; RVG, Rabes Viral Glycopeptide.

### Approaches for cargos loading into exosome

Besides the carriers of natural exosome contents, the therapeutic agents can be encapsulated within exosomes and transported to target cells for therapeutic applications (42). Various contents, including genetic material, low molecular weight therapeutics, and proteins packaged in exosomes, manifest their therapeutic impacts upon transport into the target cells (43). Several methods have been established to explain how these molecules are loaded into exosomes. There are two primary classifications (1) exogenous loading and (2) endogenous loading (which occurs during exosome formation) (44). Table 2 summarizes all the exosome drug-loading strategies with their advantages and disadvantages (48).

**Table 2:** A comparison of various methods for loading cargo into exosomes.

**Loading category**	**Loading method**	**Procedure**	**Advantages**	**Disadvantages**	**References**
Exogenous-active	Electroporation	Exosome (roughly 20 μg refers to protein weight) μL buffer electroporation at 400 V in a total of 200 adjusting to specific nuclei acid transfection machine	Relatively higher efficiency, appropriate for some macromolecules, and inexpensive. Limited impact on the ligands and receptors presents on the membrane surface of exosomes	May cause exosomes/therapeutic siRNA to aggregate and alter their morphological properties	(45)
	Sonication	Sonication is carried out repeatedly for a set period (in seconds) while resting on ice.	Cheap and common	Limited drug loading it may affect the quality of the exosome's membrane.	(46)
	Freeze/thaw	Frozen quickly at −80°C and repeatedly thawed	Simple and inexpensive	Exosomes can have low efficiency, structural damage, and aggregation issues.	(47)
	Saponin-mediated permeabilization	Exosomes can create pores when expressed to chemicals like saponin	Higher efficiency Simple and easy. Increased loading efficiency with no change in the exosomes size or surface charge	May jeopardize exosome integrity and alter their immunogenicity. Toxicity and hemolysis risks if not washed.	(47)
	Hypotonic dialysis	Drug-containing exosomes can be obtained by stirring a mixture of exosomes and therapeutics on the dialysis membrane, which is then placed into dialysis tubes.	Simple operation	Loading limitation for proteins and peptides and limited cellular uptake efficiency.	(48)
Exogenous-passive	Simple drug incubation	Incubate desired cargos with exosomes and allow cargos to diffuse via a concentration gradient.	Simple operation and less exosome integrity destruction.	Limited loading efficiency	(48)
Endogenous	Incubation in the donor cell	The donor cells are treated with a therapeutic, producing exosomes containing the therapeutic.	Enhancement of encapsulation efficiency	Poor purity	(49)
	Transfection	Lipofectamine 2000, a transfection reagent. Aids exosome fusion	Large cargo friendly and more efficient	Risks of toxicity, expensive, and could compromise cargo quality.	(50)
	Liposome-mediated method	Exogenous substances are packed in the exosomes by modifying the parental cells via liposomes.	Enhancement of encapsulation efficiency	Incomplete evaluation	(51)
	Membrane protein engineering methods	A packaging protein-bound cargo RNA is transfected into the donor cell. Exosomes with the protein-bound RNA is secreted by the donor cell to target specific cell.	Specifically (cargo)	Exosome deterioration	(52)
	Cell extrusion	Cargo loading can be achieved using a syringe extruder with a filter, which applies mechanical force to the exosome membrane.	Efficient and relatively simple	Mechanical stress can lead to exosome membrane damage and loss of content. Exosome impurity and loss are more likely to occur.	(47)

siRNA, Small interfering RNA.

#### Exogenous loading

Exogenous (Direct loading) approaches are used to load cargo into exosomes after they have been isolated (53). Exogenous Loading methods include sonication, electroporation, and incubation. Others are freeze/thaw, saponin-mediated permeabilization, and extrusion are uncommon for loading siRNA, miRNA, protein, bioactive components, and anticancer drugs into exosomes. This loading technique can be categorized into two distinct types: passive loading and active loading. However, they can also be combined to provide the best possible loading efficiency (50). According to its name, the passive exogenous involves the passive incubation of the cargos with the exosomes. Examples of drugs passively loaded into the exosomes include paclitaxel, doxorubicin, and curcumin (54). Using cholesterol conjugates is another example that improves exosome entrapment efficiency by covalently attaching cholesterol to the drug (55). Active exogenous, in contrast to passive ones, involves rupturing the exosome membrane via electroporation, sonication, saponin-mediated permeabilization, and hypotonic dialysis to allow cargo entry into the exosomes (56). The frequently employed approach in this subclass is electroporation, which uses electrical current to create pores in the membrane for encapsulating therapeutic agents into exosomes, including nucleotides (48).

#### Endogenous loading

Endogenous exosome loading refers to introducing particular therapeutic agents before exosome secretion or, more specifically, at the exosome biogenesis stage. It is a method of modifying donor cells, also known as cell engineering. Exosomes containing therapeutics can be secreted by donor cells (57). A Passive endogenous loading can be achieved by transfecting a plasmid or directly inserting the oligonucleotide (mRNA, miRNA, or shRNA) into the receiving cell. This approach relies on the cell's natural mechanisms for gene expression and does not involve external entities to enhance the uptake of molecules. Targeted and modular exosome loading (TAMEL) is a method of active endogenous loading achieved through the engineering of exosomes, allowing for the precise delivery of the cargo to designated cells or tissues (48). Active endogenous loading may also be performed via cell extrusion, a combination of viral capsid 9AAV vector and exosomes, a liposome-based technique, and a transmembrane protein engineering approach to deliver cargo into donor cells.

### Targeting brain with genetically modified exosome

Exosomes play an essential function in brain biology. Neural exosomes are small vesicles released by neurons and contain various biomolecules. Neurotransmitters and glutamate stimulate the production of exosomes, then exosomes deliver their contents to nearby and distant brain cells, regulating their function and health (58). The exosome is a great choice or application for supplying desired cargo to the brain because of its naturally existing biological function and the availability of receptor-friendly proteins on its surface. However, it is essential to engineer exosomes to express proteins or ligands with stronger affinity for their brain target cells, following receptor/ligand phenomenon (59, 60). Exosome surface modification also reduces the uptake of exosomes in off-target areas, including the liver, kidney, spleen, etc. Some exosomes have a natural homing capability to deliver therapeutics to target regions. For instance, in one study, Perets et al. (61) used X-ray computed tomography (CT) with gold nanoparticles to see how exosomes migrate throughout the brain. Researchers discovered that exosomes from bone marrow mesenchymal stem cells (MSC-exo) assemble particularly in affected regions up to 96 h after administration in distinct brain disease models. Exosomes disseminate within 24 h in healthy brains. According to the study, neuro-inflammatory signals and exosome accumulation in pathological brains are related, indicating an inflammatory-driven homing mechanism. Exosomes are selectively taken up by neuronal cells in specific pathological locations, highlighting the possibility for targeted therapeutics delivery in brain diseases. In another study, effects of status epilepticus (SE) on mice's hippocampus injury were examined by Long et al. (62), who investigated the intranasal delivery of anti-inflammatory A1-exosomes produced from human bone marrow-derived mesenchymal stem cells. Within 6 h, A1-exosomes arrived in the hippocampal region, lowering inflammation and neuronal death. The rats who receive treatment show improvements in memory, neurogenesis, and cognitive function while the control group shows declines. This shows that A1-exosome administration via intranasal route effectively mitigates SE-induced hippocampus damage and avoids cognitive deficits.

**Figure 3:**
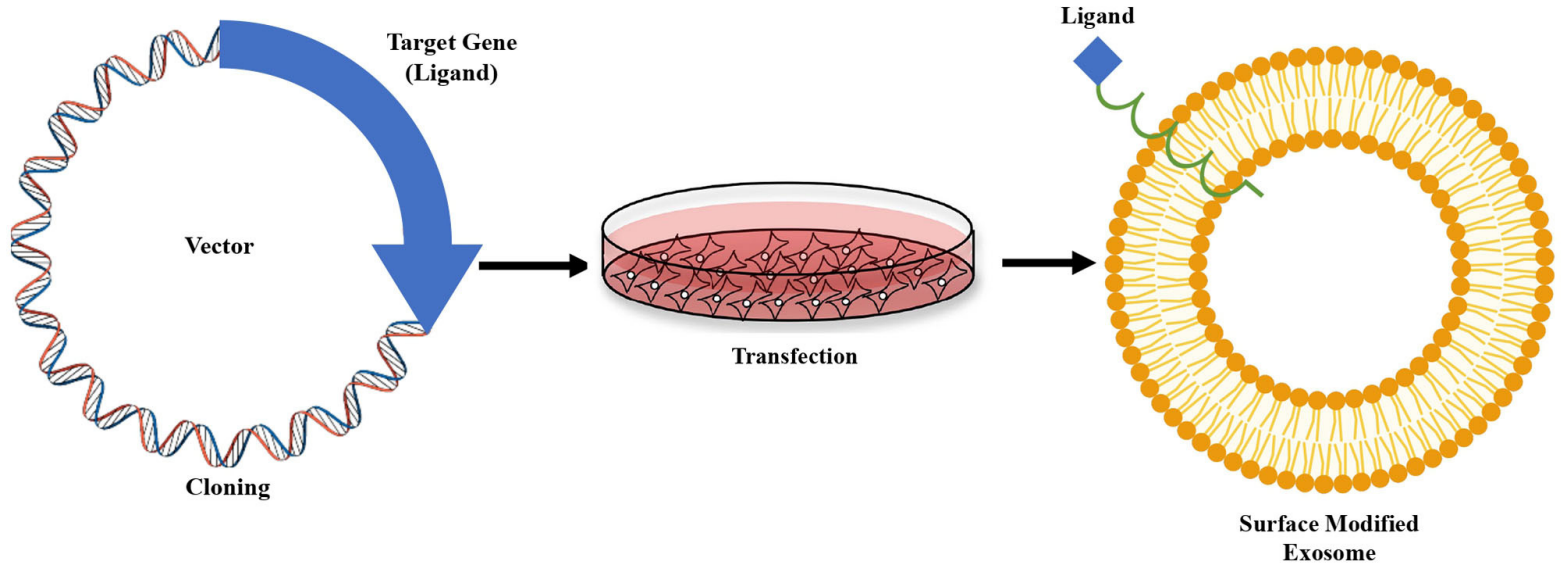
Genetic modification strategy of exosome. Sequence of a receptor specific “ligand” fused with sequence of any exosome's surface protein, are then cloned into a vector. The vector is then transfected into cultured cells. Isolated exosomes from the cells carry the ligand attached to the surface protein.

This paper discusses only exosome modification by genetic engineering method for brain-targeted therapeutic delivery. Genetic engineering strategies are most commonly used to address several shortcomings of naked exosomes and improve their ability to function as CNS drug delivery vehicles. It involves fusing the desired peptide sequence or target gene ligand with an exosomal membrane protein that is displayed on exosome surface, as shown in Figure 3 (30). Lamp2b-RVG is the most often employed hybrid plasmid for genetically engineered brain-targeting exosome modification. Lamp2b is a highly expressed membrane-localized exosomal protein extensively used with a targeting sequence. Lamp2b is a member of the protein family called lysosome-associated membrane protein (LAMP), primarily distributed within lysosomes and endosomes. Researchers established that the targeting sequence might be added to the N-terminus of the lamp2b extracellular domain, which is visible on the surface of the exosomes (63).

**Figure 4:**
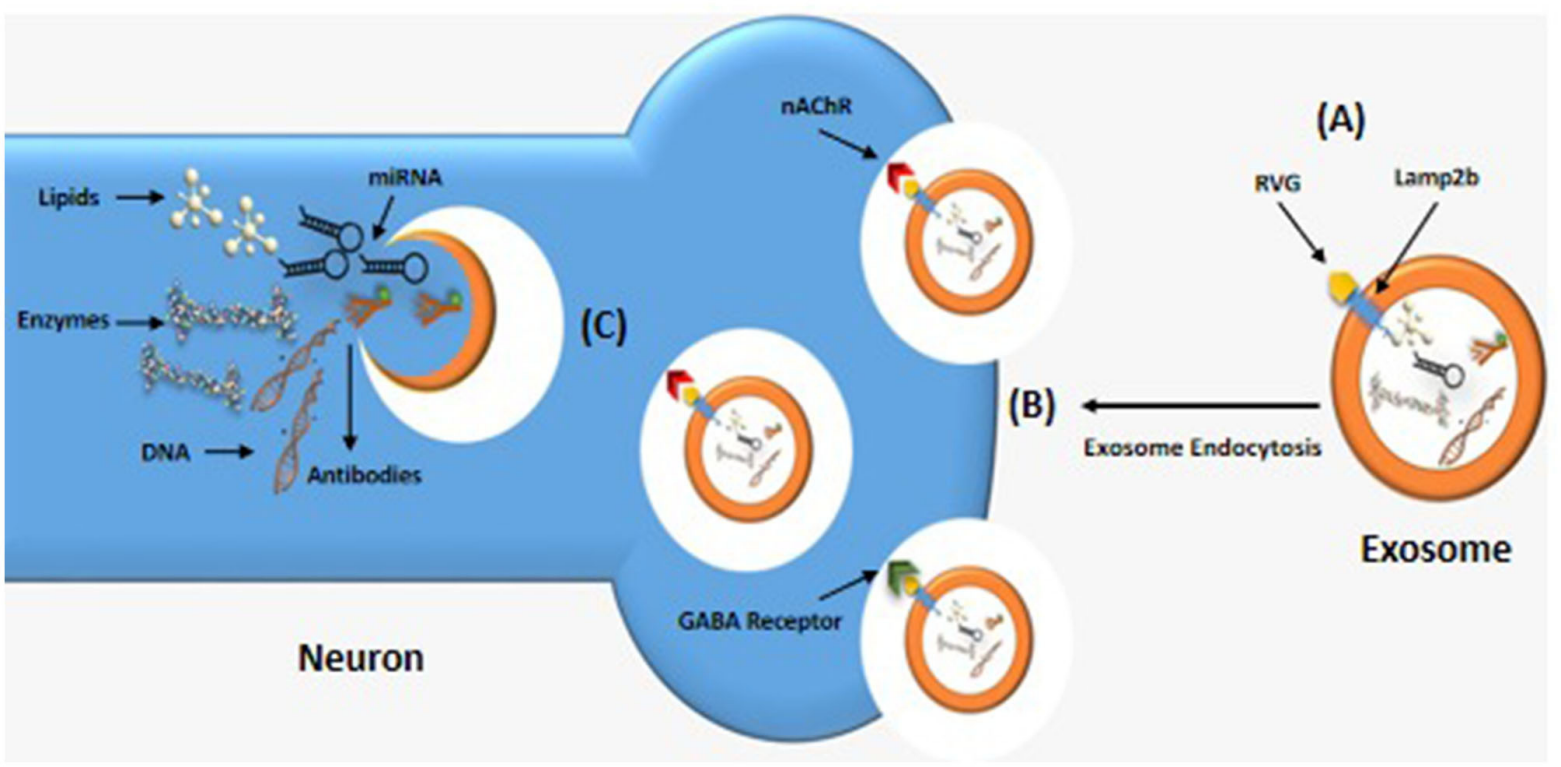
Brain-targeting exosomes. **(A)** A genetically modified exosome with RVG peptide linked to the lamp2b transmembrane protein. **(B)** RVG peptide binding to nicotinic acetylcholine (ACh) or g-aminobutyric acid (GABA) receptors, allowing the exosomes to enter neuron by endocytosis. **(C)** Inside the neuron, the exosome releases a variety of therapeutic compounds such as lipids, RNA, and DNA.

The most well-known brain-targeting peptide is rabies virus glycoprotein (RVG) which serves as a ligand to bind the nicotine acetylcholine receptor (nAChR) or g-aminobutyric acid (GABA) receptor (64), present on both neuron and brain endothelial cells, as shown in Figure 4. RVG, which has a 505 amino acid glycoprotein, is found in the lipid layer of the rabies viral surface (64). Various research shows that 29 amino acid peptides [RVG29-d9R, YTIWMPENPRPGTPCDIFTNSRGKRASNGGG (d) RRRRRRRRR] derived from RVG can transfer siRNA and cationic polymers into neural cells through cellular transduction mediated by neuronal nAChR (65). Furthermore, the amino acid sequence of rabies virus-derived peptides is the crucial nerve-binding site (66). A significant amount of data demonstrated that after being administered *in vitro*, the rabies virus-derived peptide (KSVRTWNEIIPSKGCLRVGGRCHPHVNGGGRRRRRRRRRRRRRRRRRRRRRRRRR) accurately and successfully delivered its cargo to the brain (66). Once bound, exosomes are internalized and transported across the BBB via transcytosis, allowing therapeutic compounds to be delivered into the central nervous system. This targeted delivery system is a promising approach for treating brain diseases (67).

### Advantage of exosomes as therapeutic delivery systems for brain disorders

**Figure 5:**
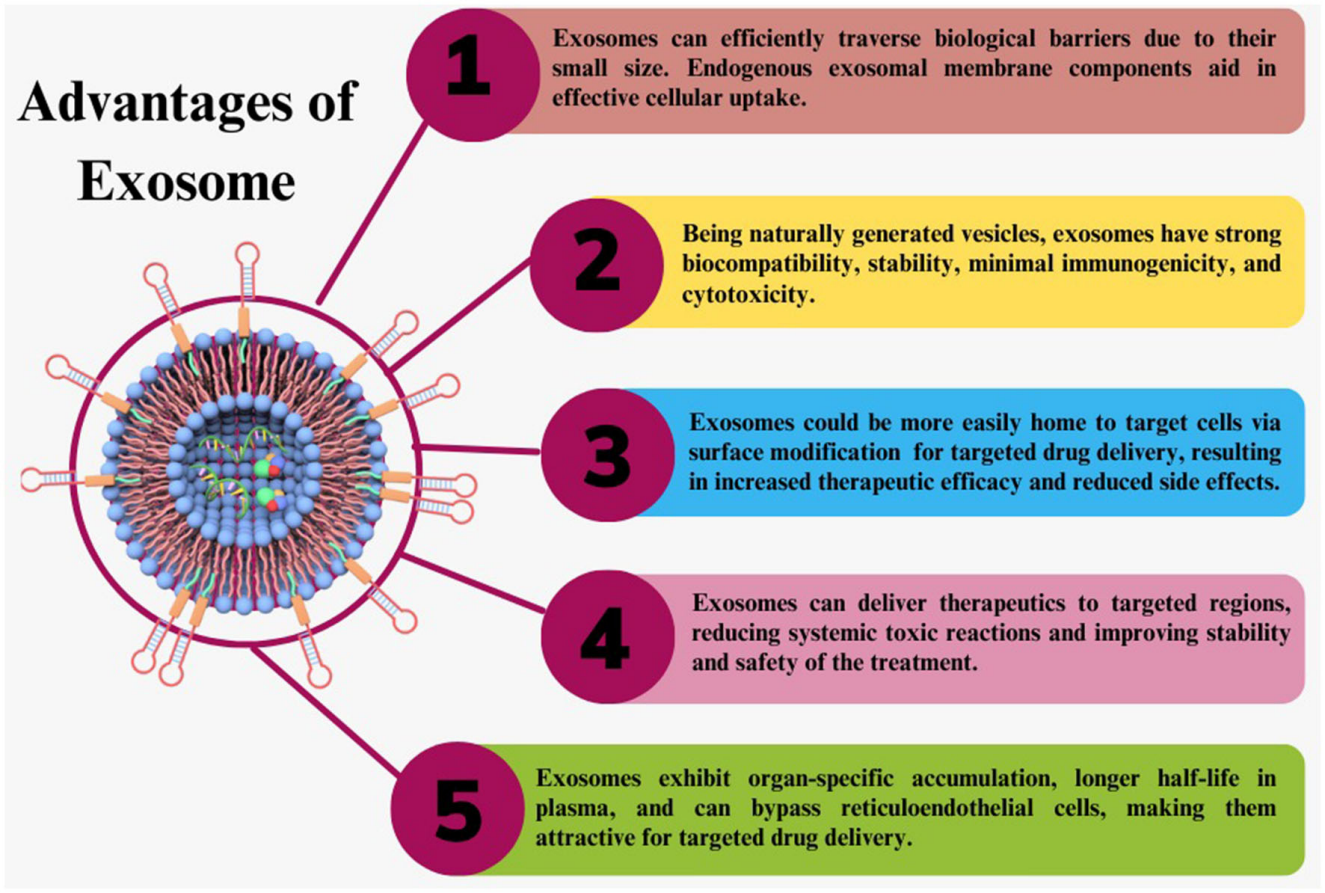
Exosome advantages. Exosomes have many advantages over synthetic therapeutic delivery methods as a naturally occurring carrier for therapeutic delivery.

Exosomes being natural biomolecules, have low immunogenicity, prolonged half-life in the bloodstream, biocompatibility, ability to cross BBB, and lower cytotoxicity. They are a safe and effective option for delivering therapeutic cargo to the brain (68). Exosomes can transport both hydrophilic and hydrophobic drugs with excellent efficiency. Exosomes can express the T-cell marker, such as a cluster of differentiation 43 (CD43), to mimic the surface of T-cells. CD43 expression is thus a helpful defense mechanism against immune systems for exosomes (69). Brain disorders such as neurodegenerative disease, stroke, malignancies, etc., are the leading causes of disability worldwide. There are numerous challenges in treating these disorders. The main obstacle to effective therapeutic transport into the brain is the BBB presence. As elaborated in Figure 5, exosomes offer an essential advantage in bypassing the BBB due to their small size and natural characteristics. According to recent findings, exosomes can penetrate or bypass the BBB *in vivo* and *in-vitro*, with or without surface manipulation (70).

### Exosome to cross BBB

Crossing the BBB is essential for therapeutic drug delivery to the brain. The ability of exosomes to cross the BBB has received extensive research. Natural exosomes lack precise targeting and cannot travel through the BBB. To solve these shortcomings, modifications such as targeting peptides or ligands and genetic engineering techniques can be applied, although these techniques are time-consuming. The RVG peptide was employed to modify the exosomes for targeted delivery to the brain (71), as already described above. Studies have shown that when cells uptake exosomes, it can change the genes involved in tight junctions and lead to changes in the mRNA. This can increase cell membrane permeability and decrease trans-endothelial electrical resistance (72).

**Figure 6:**
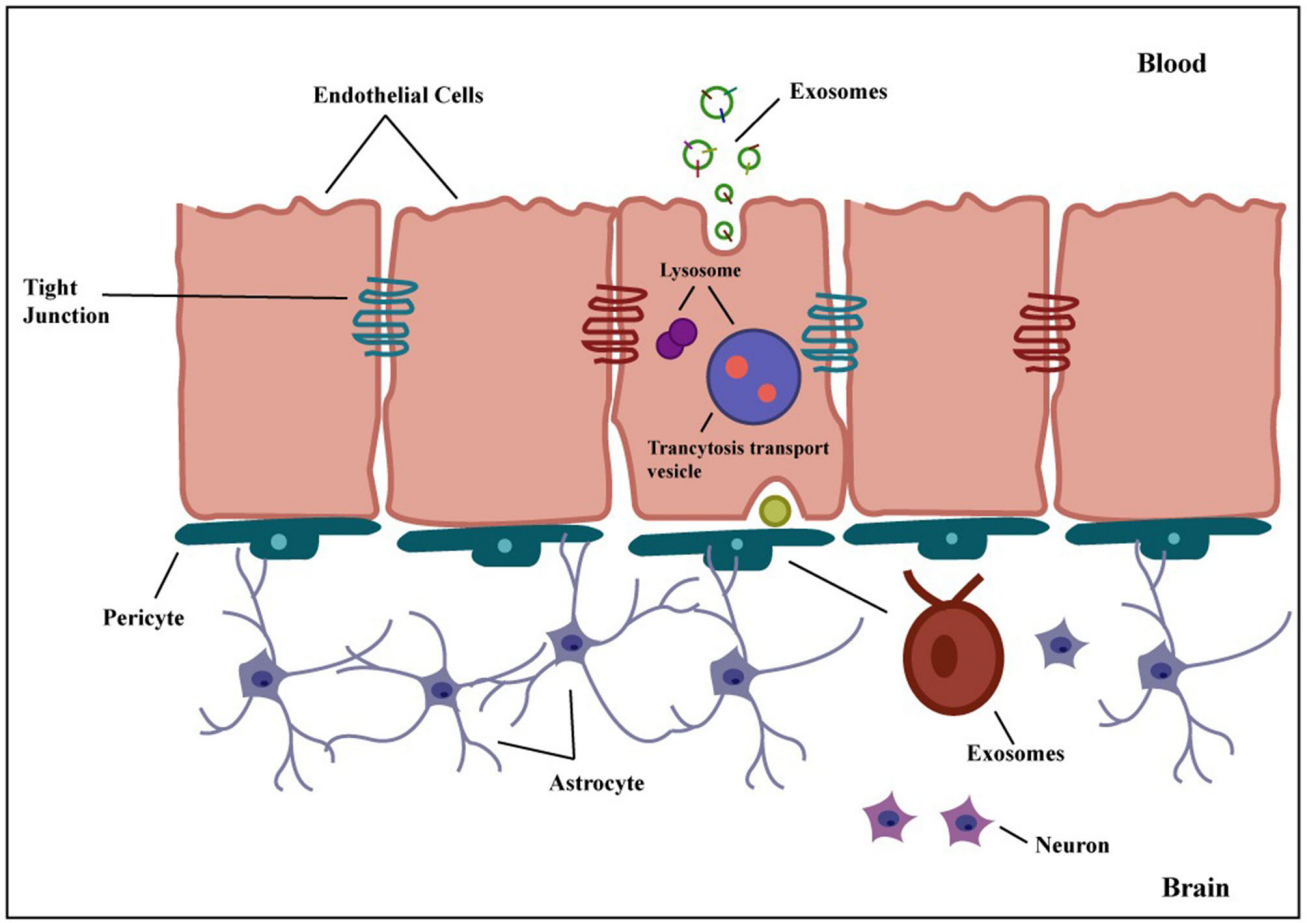
Transcytosis of exosome across the BBB. Exosomes can be genetically modified to express proteins on their surface, allowing them to attach to BBB receptors. They penetrate the BBB via transcytosis after being taken up internally by brain endothelial cells. Exosomes are released into the brain parenchyma once they have crossed the BBB, where they might interact with glial cells and neurons. Exosomes are thus a potential candidate for targeted therapeutic delivery to the brain to treat many different brain disorders.

The following mechanisms can be used to cross the BBB: receptor-mediated transcytosis (RMT), efflux, carrier-mediated transport, adsorptive-mediated transcytosis, paracellular transport, and diffusion (73). The transcytosis mechanism, shown in Figure 6, is currently the most widely approved hypothesis to explain how exosomes cross the BBB (74). Transcytosis is observed in many different types of cells, including brain cells, intestines, osteoclasts, and endothelial cells. In this process, molecules are taken up from a single side of the cell, transported across the cytoplasm, and then released on the opposite side. The movement of macromolecules from the apical to the basolateral plasma membrane is called unidirectional transcytosis in polarized cells. The initial step of these mechanisms may include absorptive (charge-dependent) or receptor-mediated transcytosis (RMT). Substances with positive charges, such as polymers, cationic lipids, albumin, nanoparticles, and exosomes, can attach to the negatively charged cell membranes and enter the cell via absorptive endocytosis (75). Although it is believed that RMT is reduced in brain endothelial cells, this process is seen in almost all endothelial cells (73). Recent imaging techniques have enabled extensive studies of the transcytosis process in brain endothelial cells (76). RMT can be caused by a variety of receptors in the BBB, including the insulin receptor, lipoprotein transport receptors, and transferrin receptor. However, it does not express some receptors, such as albumin receptors (73). The vesicular system regulates the internal movement of molecules. Three types of endocytic vesicles have been found in brain endothelial cells: macropinocytotic vesicles, absorptive-mediated endocytosis of extracellular molecules, and clathrin-coated (77). Clathrin-coated vesicles are membrane-bounded compartments that play a crucial role in many internalization processes, facilitated via about 20 distinct receptors in endothelial cells of the BBB. After the internalization of an exosome, it undergoes an intracellular sorting process and is transported to an early endosome. Endocytosis takes place in the specialized endothelial cells of the BBB at both the apical (facing the brain) and basolateral (facing the blood) membranes, resulting in the production of early endosomes specific to each process. In polarized cells, early or recycling endosomes can route back to the plasma membrane (73). In an alternative pathway, components of the vesicle formed during endocytosis can be transported to late endosomes, which have matured and acquired specific proteins and then directed to lysosomes for breakdown (78). In general, exosomes in the peripheral circulation typically have to initially traverse tightly adherent endothelial cells of the brain microvascular before transcytosis can occur and deliver them to the brain. Exosomes then engage with the pericytes and astrocytes, the subsequent two cell layers. These successive transfers reveal mechanisms for selective recognition, trans-cellular transport, and release (79).

## Application of modified exosomal therapeutics delivery in brain

Exosomes have been used extensively as nanocarriers for brain therapeutics delivery, and many studies on various brain diseases animal models have been conducted to evaluate the effectiveness of exosomal therapeutics delivery systems. These studies have focused on optimizing the delivery system by designing specific targeting peptides and utilizing different drug-loading techniques. Modified exosome potential for treating brain diseases is discussed in Table 3.

**Table 3:** The therapeutic application of modified exosomes in brain diseases.

**Brain diseases**	**Donor cell**	**Therapeutic loading**	**Modification strategy**	**Target cell**	**Function**	**References**
Alzheimer's disease	Endogenous dendritic cells	BACE1 siRNA	Lamp2b-RVG	Neuron, microglia, and oligodendrocytes	Treatment for Alzheimer's disease	(80)
Parkinson's disease	Dendritic cells	shRNA-MCs	Lamp2b-RVG	unidentified	Parkinson's disease treatment	(81)
	HEK293T	DNA aptamer	Lamp2b-RVG	Microglia neurons and astrocytes	Parkinson's disease treatment	(28)
	Primary dendritic cells	Alpha-syn siRNA	Lamp2b-RVG	unidentified	Parkinson's disease treatment	(82)
Stroke	BMSCs	miR-193b-3p	Lamp2b-RVG	Unidentified	Reduce inflammation after subarachnoid hemorrhagic	(83)
	BMSCs	miRNA-124	Lamp2b-RVG	Cortical neural progenitor	Promote neurogenesis after ischemia	(84)
Brain cancer	MSC	Antisense oligonucleotide against miR-21	Lamp2b-T7	Glial cells	Brain cancer treatment	(85)
ZIKV infection	HEK293T	ZIKV-specific siRNA	Lamp2b-RVG	Microglia, neurons, and astrocytes	Antiviral therapy	(25)
Addiction (morphine relapse)	HEK293T	Mu siRNA	Lamp2b-RVG	Neuro2A	Morphine addiction therapy	(85)

shRNA-MCs, short hairpin RNAs-minicircles; BACE1, beta-site amyloid precursor protein cleaving enzyme 1; ZIKV, Zika virus; MOR, morphine opioid receptors; Lamp2b-RVG, lysosome-associated membrane glycoprotein 2b (Lamp2b)-rabies virus glycoprotein (RVG).

### Alzheimer's disease

The most prevalent form of dementia, Alzheimer's disease (AD), is an incurable, degenerative neurological condition that impairs physical and mental health (86). There are no effective treatments for AD, and current treatments are confined to controlling symptoms and offering temporary comfort rather than slowing or preventing the illness's progression. Medications such as cholinesterase inhibitors and memantine can help improve cognitive performance. Still, they fail to treat the fundamental causes of the disease, such as the formation of amyloid plaques and neurofibrillary tangles in the brain (39). Furthermore, the effects of these drugs differ from person to person, and their benefits fade over time. They also have adverse side effects (87). There is continuing research to create treatments for AD that address its fundamental causes by delivering therapeutic medicines to target locations via exosomes. These novel techniques can potentially provide more effective interventions to treat AD while also giving long-term benefits to patients.

AD is caused by the accumulation of specific proteins, such as C-terminal fragments and amyloid beta peptide (Aβ) produced from amyloid precursor protein (APP) (88). Proteases such as beta-site amyloid precursor protein cleaving enzyme 1 (BACE1) and γ-secretase play critical roles in the breakdown of APP, leading to Aβ peptide production (89). As increased BACE1 activity is connected to dementia, inhibiting or reducing BACE1 activity could aid AD therapy (90). However, blocking γ -secretase might have unfavorable effects due to its vital role. BACE1 may be a feasible target because studies on BACE1 knockout in mice had not revealed any adverse consequences (91).

Alvarez-Erviti et al. investigated how exosome-mediated delivery of siRNAs to inhibit BACE1 expression could improve the neuropathological state in mice with Alzheimer's disease (AD) (80). To construct genetic engineer-targeted exosomes, a plasmid containing RVG peptide-linked lamp2b was transfected into self-derived dendritic cells. The isolated modified exosomes are loaded with therapeutic cargo, such as GAPDH siRNA. When injected intravenously into mice, they successfully conveyed the cargo to the brain, reducing amyloid production and neurodegeneration associated with AD (92).

Specific miRNAs, particularly the miR-29 family, which is involved in neuron homeostasis, are dysregulated in the brains of AD patients (93). According to a study, expression BACE1 is raised when the miR-29 family is diminished in AD. To combat this, Jahangard et al. created modified exosomes targeting BACE1 packaged miR-29 (94). Exosomes packaged with either miR-29a or miR-29b were produced in rat bone marrow mesenchymal cells and HEK293T cells. In U87 cells, the exosomes encapsulating miR-29b significantly decreased BACE1 expression relative to the control group. When a rat model of Alzheimer's disease was bilaterally administered with these exosomes, improved learning capacity and blocked the amyloidogenic process was observed.

In another study, the ability of mesenchymal stem cell-derived exosomes with or without RVG surface engineering was investigated to correct memory impairments by regulating inflammatory responses in an AD animal model. Both of these reduced plaque deposition and Aβ concentration in hippocampus and cortex. Although surface engineered exosomes showed enhanced activity (95). In a similar study, adipose-derived stem cells derived RVG expressing exosomes enriched in neprilysin derivative facilitated the breakdown of Aβ plaques. When administered, these engineered exosomes increased anti-inflammatory cytokines and decreased pro-inflammatory cytokines in the hippocampus. This precise targeting and regulation of inflammation showed a promising treatment for AD (96).

### Parkinson's disease

Parkinson's disease (PD) is the most prevalent synucleinopathy. It is caused by a progressive loss of dopaminergic neurons in the substantia nigra pars (97). Excess buildup of misfolded and aggregated alpha-synuclein protein in neurons can result in the onset of a Lewy body and the loss of dopamine-producing neurons, which results in cell death and common psychological and motor impairments. These are the critical indicators of pathogenesis (98). PDs are currently being treated with drugs, surgery, and therapy. Levodopa and dopamine agonists are two examples of medications that can aid symptom management but can have potentially harmful effects (99). Deep brain stimulation (DBS) surgery aims to alleviate symptoms by implanting electrodes in the brain's cortex. Moreover, physical and speech therapy can help with movement and communication. While these treatments can help manage symptoms, there is no curative for Parkinson's disease, and ongoing research aims to develop new and more effective remedies. As a result, one option for PD treatment is to reduce alpha-synuclein protofibril aggregation (100). Targeting the pathogenic alpha-synuclein aggregates that are the hallmark of Parkinson's disease (PD) using modified exosomes is one potential therapy under study.

Cooper et al. has demonstrated the effectiveness of modified exosomes for delivering alpha-synuclein siRNA to lower total and aggregated alpha-synuclein levels in mice brains. siRNA-loaded exosomes expressing RVG were delivered into mouse brains. The RVG exosomes enabled the systemic delivery of siRNA to the brain, resulting in an abrupt reduction in the levels of alpha-synuclein mRNA and protein in the brain. Additionally, the treatment reduced the buildup of intraneuronal proteins, especially in the dopaminergic neurons of the substantia nigra (82).

Nevertheless, the short-term effectiveness of siRNA intervention limits its success. However, fresh research by Izco et al. has demonstrated the possibility for long-term efficacy using short hairpin minicircles (shRNA-MCs) packaged within the same modified RVG-exosomes, to target and decrease α-syn aggregation. These shRNA-loaded RVG exosomes were administered intravenously to the brains of Parkinson's disease rats (81).

In a different study, Ren et al. found that aptamers delivered by RVG-exosomes to neurons *in vitro* and PD models lessened the pathogenic aggregates caused by alpha-syn pre-formed fibers (PFFs) and alleviated the mice's behavioral impairments. The aptamer, F5R1, works as a chemical antibody that targets alpha-synuclein selectively. In cultured brain cells, an F5R1 aptamer loaded into RVG-exosomes efficiently inhibited alpha-synuclein aggregation and reduced cell abnormalities and mitochondrial dysfunction. When given to mice with alpha-synuclein-induced problems, these modified exosomes drastically reduced alpha-syn aggregates in the brain and restore motor function. This study reveals the potential of employing modified exosomes for the targeted delivery of aptamers as a promising strategy to counteract protein aggregation in brain diseases (101).

### Stroke

A stroke, often known as a brain attack, is caused by an abrupt rupture of a blood vessel in the brain or an obstruction of blood flow to the brain, and it can be either ischemic or hemorrhagic (102). About 75% of survivors are disabled, making it one of the most severe life-threatening neurological conditions. miRNAs in circulation have a vital role in regulating brain development and function and controlling numerous aspects of neurological impairment (103). The therapeutic role of miRNA in a variety of CNS illnesses has been demonstrated, and it is considered to entail neuroinflammation control (104). Two studies investigated the therapeutic potential of modified exosomes for brain diseases.

Hemorrhagic stroke frequently results from subarachnoid hemorrhage (99). An essential factor in developing subarachnoid hemorrhage is inflammation (105). In one study by Lai et al. (83), modified exosomes were utilized to deliver miR-193b-3p in mice with subarachnoid hemorrhage. The RVG exosomes separated from bone marrow stem cell cultures, were electroporated with the miR-193b-3p. The RVG/Exos/miR-193b-3p was then injected into the brains of SAH mice via peripheral injection. The *in-vivo* investigations demonstrated that administering RVG/Exos/miR-193b-3p efficiently reduced the levels of Histone deacetylase 3 (HDAC3). HDAC3 has been linked to neuroinflammation, and suppressing its expression and activity can protect the brain. The study additionally looked into the effects of miR-193b-3p treatment on many SAH-related aspects, including neurological scores, brain fluid content, BBB integrity, and neurodevelopmental issues. The findings showed that giving miR-193b-3p via modified exosomes diminished the expression of inflammatory cytokines, which decreased neurodegeneration, brain edema, BBB damage, neuroinflammation, and neurodevelopmental issues associated with SAH. The results of the *in-vivo* experiments revealed that administering RVG/Exos/miR-193b-3p effectively reduced the levels of HDAC3, a protein associated with inflammation, in various regions of the brain. HDAC3 plays a role in neuroinflammation, and suppressing its expression and activity can protect the brain. The study also assessed the impact of miR-193b-3p therapy on various aspects of SAH, such as neurological scores, brain fluid content, BBB integrity, and neurodevelopmental problems.

Ischemic stroke is caused by blood flow and oxygen disruption to the brain, the most common type of stroke (106). miRNAs are essential in regulating disease and brain growth in the neurological domain. miRNAs experience dysregulation during ischemia, which results in long-term neuronal damage. Regulating these miRNAs to target particular proteins involved in the injury process could be a therapeutic approach to reduce neuronal damage following brain ischemia (107). One of these, miRNA-124, has significant expression in the brain and is essential for proper brain development (108). According to studies, miR-124 is dysregulated following cerebral ischemia, which impairs neuronal differentiation (109). Yang et al. (84) explored, using modified RVG-exosomes for delivering miR-124 promoted neurogenesis after ischemia. These exosomes loaded with miR-124 stimulated cortical neuro progenitor cells to differentiate into neurons at the site of brain damage caused by ischemia, promoting extensive cortical neurogenesis and protecting against ischemic damage. This study highlights the potential of RVG-exosomes as a therapeutic approach for promoting brain repair and recovery after ischemic stroke.

### Brain cancer

Gliomas are the most common intracranial tumors among individuals diagnosed with cancer and are found in the glial tissue of the CNS. These tumors constitute around 32% of all brain and CNS tumors, with 80% being malignant (110, 111). Progenitor cells or neuroglial stem cells are the primary sources of origin for glioblastomas, which comprise most newly discovered gliomas. Glioblastoma patients typically have a short lifespan, with an average of 1 year, and only a tiny proportion (5%) survive for more than 5 years (112). Gliomas face significant treatment challenges due to the disease's diffuse nature and innate barriers within the brain. Gliomas invade the brain tissue surrounding them, making surgery more difficult and increasing the chance of recurrence (113). The brain's susceptibility to radiation limits the efficiency of radiotherapy, whereas chemotherapy faces barriers such as the blood-brain tumor barrier and BBB, which restrict drugs from reaching tumor locations (114).

Additionally, some drugs used in glioblastoma treatment, like temozolomide (TMZ), can have severe toxicity and adverse side effects (115). Even after undergoing complete surgical resection and receiving additional treatment with chemotherapy and radiation, the glioma tumor is likely to reappear within 8 months (116).

One promising miRNA for glioblastoma treatment is miR-21, which is significantly elevated in glioblastoma tumors and promotes cancer growth by inhibiting the synthesis of the PTEN protein (Tumor Suppressor) (117). To offset this impact, miR-21 was knocked down to reduce tumor cell proliferation and boost apoptosis. In the case of glioblastoma gene therapy, a glioma-specific ligand is required instead of RVG on the surface of exosomes. Glioblastoma cells have a high level of the cellular transferrin receptor (TfR), making it an appealing target for gene delivery. T7, a TfR-binding peptide, is an appropriate ligand for glioblastoma-targeted administration. Kim et al. (85) created T7-exosomes to transport AMO-21 to the brain, which inhibits miR-21. T7-Exo succeeded more than unmodified and RVG-decorated exosomes in delivering AMO-21 to glioblastoma cells and reducing tumor sizes *in vitro* and *in vivo* investigations. The outcomes also involved a rise in the expression of Phosphatase and tensin homolog (PTEN) and Programmed cell death 4 (Pdcd4) proteins and a reduction in tumor size. This study emphasizes the potential of T7-Exo as a successful vehicle for the targeted delivery of therapeutic drugs in managing glioblastoma. However, more investigation is required to confirm and improve these findings before applying this strategy in clinical settings.

### Drug addiction

Drug addiction is a recurring, long-term condition induced by repeated drug exposure in the brain, which alters the brain's reward system (118). A significant portion of the population suffers from drug addiction, which causes enormous social, medical, and economic problems (119). Despite an increased understanding of the brain changes underlying addiction, current treatments have limitations due to their non-specific effects and poor outcomes. One reason is the brain's ability to prevent the entry of non-targeted therapies. Exosomes have been explored for treating drug addiction because they can effectively reach the brain (120). The mu-opioid (MOR) receptor greatly influences opioids' addictive properties, and it has been suggested that treating opioid addiction by explicitly targeting the MOR could be effective (121). According to studies, rats without the MOR receptor do not experience the pleasurable benefits of opioids, and MOR antagonists have shown promise in reducing the risk of relapse. Based on these findings, the MOR gene has been chosen as a potential drug addiction treatment target (122).

For the cure of morphine addiction, Liu et al. (123) constructed RVG-modified exosomes that were loaded with MOR siRNA and delivered to neuro2A cells and the mouse brain. This leads to a significant decrease in MOR mRNA and protein levels and positively lowered the chance of morphine relapse.

### Brain viral infection

Viruses invade host cells and multiply within the organism through a variety of processes. The ZIKA virus (ZIKV), a flavivirus, can traverse both the placenta and the BBB and infect the developing fetal brain. This can result in fetal microcephaly, a severe neurological condition (124). ZIKV linked to neurological diseases, threatens global health (125). Few therapeutics mainly target brain tissue; most are incredibly toxic and do not successfully traverse BBB. No treatment or vaccine is specific to the ZIKA virus currently available (126). Although gene silencing therapies based on oligonucleotides have shown distinct advantages in therapeutic situations, transferring nucleic acid into brain cells is still tricky. Zhang et al. (127) aimed to develop a targeted antiviral therapy to treat symptoms of ZIKV-induced microcephaly that could overcome the placental and BBB obstacles. To achieve this, they designed RVG-modified exosomes that contained antiviral siRNA capable of inhibiting ZIKV infection. These RVG-engineered siRNA-loaded exosomes were injected into pregnant AG6 mice intravenously, allowing them to penetrate the placental barrier and reach the fetal mice from the mother's circulation. Exosomes could pass across the fetal BBB and target the brain. The result showed that intravenous administration of RVG-siRNA exosomes could shield fetuses against ZIKV infection and lessen neuroinflammatory response and viral-associated neurological symptoms in the brain.

## Clinical exosome trials

A survey on ClinicalTrials.gov shows the major applications of exosomes are biomarkers, exosome-therapy, drug delivery systems, and cancer vaccines. Exosomes from plant cells, mesenchymal cells, T cells, and dendritic cells are used for the treatment of different diseases. In addition, exosomes from these sources are promising carriers for drug delivery systems. Exosomes derived mainly from dendritic and mesenchymal cells are used as drug delivery vehicles, which have applications in neurological illness, inflammation, and cancer. In the direct method, exosomes are loaded with therapeutic agents, while through indirect methods, proper cells are genetically engineered or co-cultured with therapeutic agents to produce artificial exosomes (128). Over the past few years, research on clinical applications utilizing exosome technology as drug delivery vehicles has grown significantly. Exosomes gained significant attention in the field of drug delivery systems (DDS) due to their unique features and encouraging preclinical results (128). As a result, various clinical trials have been completed or are now continuing to evaluate the efficacy and safety of exosomes *in vivo* for brain disorders, either as therapeutic agents or drug delivery systems (DDSs; Table 4). A study reveals a total of 116 trials have been documented, with 58 (50%) of them being used for biomarker applications. Thirty-three (28.44%) trials have been documented in the case of exosome therapy. For drug-delivery system trials, 6 (5.17%) studies have been registered, whereas for exosomes basic analysis, 17 (14.66%) research have been reported. Finally, two clinical trials (1.72%) are dedicated to exosome vaccine research (132). Exosomes in clinical trials must adhere to good manufacturing practice (GMP). A GMP-grade exosome production technique includes the cell type, culture environment, cultivation system, and culture media (133).

**Table 4:** List of exosome-based clinical trials for brain disorders.

**Trial ID: description**	**Disorder**	**Phase**	**Outcome measures**	**Source of exosomes**	**References**
**NCT03384433:**Allogenic mesenchymal stem cell derived exosome in patients with acute ischemic stroke.	Cerebrovascular disorders	Phase 1/Phase 2	The occurrence of treatment-emergent adverse events (deteriorating stroke, stroke recurrences, cerebral edema, seizures, hemorrhagic transformation). The degree of disability experienced by stroke patients.	Mesenchymal stem cell	(129)
**NCT01550523:**Pilot immunotherapy trial for recurrent Malignant gliomas	Malignant glioma of brain	Phase 1	Evaluate MRI responses in recurrent malignant glioma patients treated with GMP AS ODN combination for safety and therapeutic impact	Glioma	(130)
**NCT04388982:** Evaluate the safety and the efficacy of exosomes derived from allogenic adipose MSC-Exos in patients with Alzheimer's disease	Alzheimer's disease	Phase 1/2	Assessing safety (adverse events, immunogenicity) and efficacy (cognitive function, functional independence, biomarkers) in Alzheimer's subjects receiving allogenic MSCs-Exos.	Allogenic adipose MSCs	(131)

## Conclusion and future perspective

Until now, transporting therapeutics to the brain has posed a long-running issue for pharmaceutical companies. On the other hand, exosomes are a promising new kind of therapy delivery vehicle compared to synthetic NP systems, as they offer superior biocompatibility and non-toxicity and do not carry any immunogenic properties. On top of that, they have homing capability, excellent stability, and can penetrate BBB. Treatment of the majority of brain illnesses is significantly hampered by the BBB's impermeability to therapeutics. Since they can pass the BBB, exosomes have been used as effective therapeutic delivery techniques to treat a range of brain diseases. For making any therapeutic delivery tool to be successful, it must be able to reach the intended site with minimal side effects. Many exosome engineering techniques have been used to efficiently administer exosomes to specific recipient cells and reduce off-target effects, such as surface modification of exosomes and peptide conjugation to exosomes.

Bioengineered exosomes, in particular, have enormous potential for developing new means of delivering pharmaceuticals and biological payloads to brain cells, such as siRNA, mRNA, proteins, and peptides. To build new treatments for brain diseases, an in-depth study is required on constructing hybrid or modified exosomes (134). Engineered exosomes that can target the brain have demonstrated promising outcomes for brain administration in preclinical research, but they also need to undergo thorough review through well-planned clinical trials (31). It is essential to emphasize the importance of continued research into exosome engineering techniques. Research efforts should be focused on advances in altering exosomes to improve their drug-loading and targeting strategies, investigating novel methods to boost cargo loading capacity that can deliver more therapeutic agents, and creating approaches for long-term stability during storage and transportation could greatly reduce current limitations. The development of imaging techniques for quantitative/qualitative tracking of exosomal therapeutics delivery to the brain parenchyma *in vivo* and identification of the specific BBB mechanism of exosomes are required to effectively produce clinically accepted exosome delivery vehicles for brain diseases. Manufacturing exosomes for therapeutic applications will need much work and should be enough for further research. The therapeutic effect may be compromised by exosome quality and purity standards, which should be improved to strengthen the therapeutic advantages of exosomes. Growing evidence has shown that exosomes containing therapeutic compounds have produced advancements in various brain diseases described above. However, to use them as brain drug delivery shuttles in clinical settings, it is important to standardize and optimize multiple aspects of the process, such as selecting the appropriate source for selection and isolation, characterizing the exosomes, loading them with drugs, targeting them to specific regions of the brain. Achieving standardization and optimization requires additional research and collaboration among experts and interdisciplinary methods to bring exosome-based therapeutic delivery from the laboratory to clinical trials, ultimately benefiting patients suffering from brain diseases.

## Author contributions

SF: Conceptualization, Methodology, Visualization, Writing—original draft, Writing—review & editing. AQ: Conceptualization, Methodology, Resources, Writing—review and editing. SA: Writing—review & editing. AH: Resources, Supervision, Validation, Writing—review & editing. SM: Formal analysis, Investigation, Supervision, Validation, Writing—review & editing.
